# Prognostic role of serum cytokines level in non-small cell lung cancer patients with anti-PD-1 and chemotherapy combined treatment

**DOI:** 10.3389/fimmu.2024.1430301

**Published:** 2024-10-22

**Authors:** Hongyu Liu, Chao Zhou, Haohua Jiang, Tianqing Chu, Runbo Zhong, Xueyan Zhang, Yinchen Shen, Baohui Han

**Affiliations:** Department of Respiratory and Critical Care Medicine, Shanghai Chest Hospital, Shanghai Jiao Tong University School of Medicine, Shanghai, China

**Keywords:** NSCLC, checkpoint inhibitor, chemotherapy, cytokine, biomarker

## Abstract

**Background:**

Chemotherapy combined with PD-1 inhibitor treatment has revolutionized the standard of care for patients with NSCLC. However, the benefit is not universal, highlighting the need for precise prediction factors. Given their relationship with the immune system and non-invasive nature, serum cytokines are potential candidates for predicting the clinical effects of chemoimmunotherapy. Our study aims to evaluate the association of serum cytokines with the prognosis of patients with NSCLC treated with chemoimmunotherapy.

**Methods:**

Levels of 10 serum cytokines were detected in 60 NSCLC patients receiving chemotherapy plus PD-1 inhibitor-based treatment. Of these, dynamic samples from 19 patients were collected at baseline and after two treatment cycles. Their association with patients’ clinicopathological characteristics, PFS and OS was described and investigated using survival analysis, cox regression and time-dependent ROC analysis. Preliminary evaluation of changes in cytokine levels associated with treatment response was conducted.

**Results:**

Patients with lower baseline levels of serum IL-6, IL-5, IL-8, TNF-α and IL-10 had longer PFS, while patients with higher levels of IL-4 had longer PFS. Patients with lower levels of serum IL-6, IL-8, IL-22, TNF-α and IL-10 had longer OS, while patients with higher levels of IL-4 had longer OS. Multivariate analysis suggested that higher IL-6 and IL-5 levels were associated with poorer PFS, and higher IL-6 levels were associated with dismal OS. Additionally, changes in serum cytokine levels could be associated with treatment response.

**Conclusion:**

Our study suggests that serum cytokines, specifically IL-6, IL-5, IL-8, TNF-α, IL-10, and IL-4, are potential prognostic factors for patients with NSCLC receiving chemotherapy plus PD-1 inhibitor treatment.

## 1 Introduction

Non-small cell lung cancer (NSCLC) remains a significant global health burden, accounting for a substantial proportion of cancer-related morbidity and mortality worldwide (1). The advent of immune checkpoint inhibitors, particularly programmed cell death protein 1 (PD-1) and programmed death-ligand 1 (PD-L1) inhibitors, has revolutionized the treatment landscape for cancer, making the combination of chemotherapy and immunotherapy a promising treatment strategy for NSCLC (2). The combination has demonstrated remarkable efficacy in a subset of patients, leading to durable responses and improved survival outcomes (3–5). For example, pembrolizumab plus chemotherapy as a first-line treatment extended the median progression-free survival (PFS) and overall survival (OS) of non-squamous NSCLC patients to 8.8 and 22 months, respectively, with a five-year OS rate of 19.4% (6). However, the response rate to this combined therapy remains around 47.6%, and patients often experience with higher frequencies of adverse events compared to monotherapy (6, 7). The benefit of combining of PD-L1 inhibitor atezolizumab and chemotherapy in PFS was also observed (mPFS, 6.3 vs. 5.6 months), but the combination did not improve OS (8). Since the clinical benefit is not universal, identifying predictive biomarkers to guide patient selection and optimize treatment strategies remains a critical challenge (9).

Currently, PD-L1 expression, detected through immunohistochemistry, is the most widely used predictive biomarker for patients receiving PD-1 inhibitor-based therapy in NSCLC. However, PD-L1 expression has several limitations in clinical practice, such as the multitude of PD-L1 antibodies, assays, scoring systems, and thresholds for positivity currently used in different studies and medical centers, making it difficult for the standardization of PD-L1 as a biomarker (10). Besides, some patients with low PD-L1 expression still benefit from immunotherapy, and its predicting value is furtherly weakened in the context of combined treatment (11).

Additionally, novel biomarkers, such as tumor mutational burden (TMB) and microsatellite instability (MSI), are being discovered as potential predictors for immunotherapy (12). As emerging biomarkers, they were faced with more challenges similar with PD-L1, such as intratumoral heterogeneity, lack of universal cutoff, and inefficacy in specific populations or treatment regimes (13, 14). Most importantly, the methods above are invasive, relying heavily on biopsy, which further limits their utility (13, 14). Thus, there is a pressing need for the development of ideal biomarkers derived from noninvasive methods, which would have a wider scope of application.

Cytokines are critical small proteins involved in cell signaling and immune regulation, which garnered attention as potential prognostic markers in present studies. Cytokines are essential signaling molecules that orchestrate immune responses by modulating cellular functions, inflammation, and tissue repair (15). They play a pivotal role in shaping the tumor microenvironment, influencing tumor growth, invasion, and metastasis (16). Cytokines can either enhance or suppress antitumor immune responses, depending on the cancer type and context (17).

In this study, we evaluated the association between baseline serum levels of 10cytokines and the survival of 60 patients with NSCLC receiving chemotherapy plus PD-1 inhibitor treatment. Among these patients, serum cytokine levels were also monitored after two treatment cycles in a subset of 19 patients for longitudinal analysis. And the CorPlex platform with minimal cross-reactivity and fg/ml sensitivity was implemented to detect the cytokines levels. Our results indicated that several serum cytokines may serve as prognostic factors for these patients, providing insight into the prognostic role of serum cytokines in this clinical setting.

## 2 Methods

### 2.1 Patients

Between March 2019 and October 2020, 60 patients with NSCLC who received chemotherapy plus PD-1 inhibitor-based treatment at Shanghai Chest Hospital were evaluated in this study. Informed consent was obtained from all patients, and the study was approved by the Institutional Review Board (IRB) of Shanghai Chest Hospital [No. KS(Y)1982].

### 2.2 Follow-up and assessment

Information of patients’ medical history such as clinical characteristics, genetic mutation status, PD-L1 expression, treatment response, time of progression and time of death was gathered through medical records and telephone inquiries or outpatient visit. The final follow-up date was December 1^st^, 2022. Treatment response after two cycles was assessed based on radiographic evidence following the RECIST 1.1 criteria (18). Progression-free survival (PFS) was defined as the time from first chemoimmunotherapy treatment to disease progression based on radiographic evidence or alive at final follow-up. Overall survival (OS) was defined as the time from first treatment to death of any cause or last follow-up.

### 2.3 Sample preparation and cytokines detection

The whole blood samples of 60 patients at baseline and 19 samples among this population after two treatment cycles were obtained. Samples were collected into serum separator tubes, left to stand for 30 minutes at room temperature, and centrifuged at 1,500 g for 15 minutes. Serums then were separated and collected into ~200ul each tube and stored at -80°C until use. The CORPLEX™ Cytokine Panel 1 (CPX) (Catalog No: 85-0329) from Quanterix, Billerica, MA, USA was utilized to examine 10 cytokines (IL-4, IL-5, IL-6, IL-8, IL-10, IL-12P70, IL-22, IFN-γ, IL-1β and TNF-α) in serum samples. This assay was designed to simultaneously detect 10 cytokines with minimal cross-reactivity and fg/ml sensitivity. The procedure was executed as per the manufacturer’s guidelines. In brief, a 12.5 μL aliquot of the serum sample was diluted four times with the kit’s sample diluent. The diluted samples and prepared calibrators were added to a 96-well microplate that had been pre-coated with capture antibodies specific to each analyte. The microplate was then incubated for a period of 2 hours. Following this, proteins that did not bind were washed off, and a biotinylated detection inhibitor reagent was added and left to react for 30 minutes. After washing off the unbound detection inhibitor, streptavidin conjugated with horseradish peroxidase (SA-HRP) was added and incubated for another 30 minutes. Post this, the microplate was washed again, and the substrate was introduced. The microplate was then imaged on the SP-X platform within a timeframe of 2 to 4 minutes. Finally, the cytokine concentrations were analyzed on the SP-X analysis platform. Briefly, the calibrators added in microplates were detected and fitted with logistic regression. Cytokine concentrations then were calculated using the regression parameters and detected signals.

### 2.5 Statistical analysis

Baseline serum cytokine levels were described using median, lower quartile and upper quartile, and were compared among clinicopathological features using Mann-Whitney U test and Kruskal-Wallis H Test in SPSS Statistics 27.0.1. Changes of serum cytokine levels were compared using Wilcoxon matched-pairs signed rank test in Graphpad Prism 10. Survival analysis and cox regression were performed using the survival R package (v3.5.8; Therneau T 2024). The determination of optimal cut-point was performed utilizing the survminer R package (v0.4.9; Kassambara A, Kosinski M, Biecek P 2021) based on maximally selected rank statistics. Time-dependent receiver operating characteristics (ROC) analysis of serum cytokine levels was conducted employing the timeROC R package (v0.4; Paul B, Jean-Francois D, Helene JG 2013). Boxplots, Survival plots and ROC plots were generated using the ggplot2 R package (v3.4.4; Hadley W 2016). Line graphs of cytokine levels were plotted with Graphpad Prism 10. Significance level was set at two-sides P < 0.05.

## 3 Results

### 3.1 Clinical and pathological characteristics

Sixty patients with NSCLC who received chemoimmunotherapy were included for analysis. The clinicopathological characteristics are shown in **Table 1**. Of the 60 patients in this study, 13 patients (21.67%) were aged under 60, and 52 patients (86.67%) were male. Thirty patients (50.00%) were confirmed with adenocarcinoma, and 25 patients (41.67%) were with squamous cell carcinoma. 38 patients (63.33%) had distant metastasis. In terms of metastasis, 15 patients (25.00%) had bone metastasis, and 5 patients (8.33%) had brain metastasis. Lung metastasis was found in 12 patients (20.00%). Pleural metastasis was found in 7 patients (11.67%), and lymph node Metastasis was found in 5 patients (8.33%). Molecular pathology results suggested that 13 patients (21.67%) carried KRAS mutations, and 3 patients (5.00%) carried EGFR mutations. Meanwhile, TP53 mutations were found in 26 patients (43.33%). On the other hand, among 60 patients, 12 patients (20.00%) had PD-L1 expression levels below 1%, and 11 patients (18.33%) had PD-L1 expression levels between 1% and 50%, and 18 patients had PD-L1 expression levels of 50% or above (30.00%). In these patients, 43 patients (71.67%) received chemotherapy plus PD-1 inhibitor-based therapy as first-line treatment, while 17 patients received the therapy as second or further lines treatment.

**Table 1:** Baseline clinical and pathological characteristics of 60 patients receiving chemoimmunotherapy.

Characteristic			N	%
Age		≤60	13	21.67%
		>60	47	78.33%
Sex		Male	52	86.67%
		Female	8	13.33%
Histology		Adenocarcinoma	30	50.00%
		Squamous cell carcinoma	25	41.67%
		Non-small cell lung cancer	5	8.33%
TNM system	T	1	7	11.67%
		2	18	30.00%
		3	4	6.67%
		4	31	51.67%
	N	0	7	11.67%
		1	0	0.00%
		2	27	45.00%
		3	25	41.67%
		X	1	1.67%
	M	0	22	36.67%
		1	38	63.33%
	Stage	I	2	3.33%
		II	0	0.00%
		III	20	33.33%
		IV	38	63.33%
Bone Metastasis		No	45	75.00%
		Yes	15	25.00%
Brain Metastasis		No	55	91.67%
		Yes	5	8.33%
Lung Metastasis		No	48	80.00%
		Yes	12	20.00%
Pleural Metastasis		No	53	88.33%
		Yes	7	11.67%
Lymph node Metastasis		No	55	91.67%
		Yes	5	8.33%
Mutation status	KRAS	No	34	56.67%
		Yes	13	21.67%
		Undetermined	13	21.67%
	EGFR	No	51	85.00%
		Yes	3	5.00%
		Undetermined	6	10.00%
	TP53	No	5	8.33%
		Yes	26	43.33%
		Undetermined	29	48.33%
PD-L1 expression		<1%	12	20.00%
		1%~50%	11	18.33%
		≥50%	18	30.00%
		Undetermined	19	31.67%
Treatment-line		1	43	71.67%
		≥2	17	28.33%
Treatment Response		CR	0	0.00%
		PR	16	26.67%
		SD	31	51.67%
		PD	13	21.67%

To evaluate the association between clinicopathological characteristics and the PFS or OS of these patients, we performed survival analysis with variables described above (**Supplementary Table S1**). In terms of PFS, our results showed that lymph node metastasis was associated with poor progression-free survival and was statistically significant (HR: 4.10 [1.51, 11.15], p = 0.005620), while high PD-L1 expression (≥ 50%) was associated with good progression-free survival (HR: 0.36 [0.15, 0.85], p = 0.020216). When it comes to OS, results suggested that distant metastasis (M staging) might indicate poor overall survival (HR: 2.68 [1.15, 6.26], p = 0.022875), and lymph node metastasis was also associated with poor overall survival (HR: 5.12 [1.58, 16.63], p = 0.006564). Meanwhile, higher tumor staging was also in association with poor overall survival (HR: 2.59 [1.15, 5.84], p = 0.021923). Similarly, high PD-L1 expression (≥ 50%) also indicated better OS (HR: 0.19 [0.06, 0.58], p = 0.003185).

We then investigated the association between baseline serum levels of 10 cytokines and the treatment response of patients. As shown in **Supplementary Figure S1**, **Supplementary Table S1**, only baseline IL-4 levels were significantly higher in patients with partial response (PR) and stable disease (SD) compared to patients with progressive disease (PD) (p = 0.025).

### 3.2 The comparison of baseline serum cytokine levels among different clinical and pathological features

To assess the differences in baseline cytokine levels from clinical and pathological perspectives, we performed statistical descriptions and analysis for these 10 cytokines (as shown in **Supplementary Table S2**). IL-5 levels were significantly higher in patients aged 60 or older (**Supplementary Figure S2A**, p = 0.001). IL-12, IL-1b and IFN-γ levels were higher in patients diagnosed with squamous cell carcinoma (**Supplementary Figures S2B-D**, IL-12: p = 0.022; IL-1b: p = 0.046; IFN-γ: p = 0.026). Moreover, there was a statistically significant difference in IL-4 levels across patients with different T stages (**Supplementary Figure S2E**, p = 0.034). Patients with bone metastasis had higher serum IL-22 levels (**Supplementary Figure S2F**, p = 0.008), and patients with brain metastasis had lower IL-4 levels (**Supplementary Figure S2G**, p = 0.025). In pleural metastatic patients, IFN-γ levels were lower (**Supplementary Figure S2H**, p = 0.026), while the levels of TNF-α were higher (**Supplementary Figure S2I**, p = 0.003) in patients with lymph node metastasis. Regarding the mutation status, patients bearing KRAS mutations had higher IL-4 levels (**Supplementary Figure S2J**, p = 0.041), and patients bearing EGFR mutations had lower IL-5 levels (**Supplementary Figure S2K**, p = 0.033). For patients with TP53 mutations, IL-1b and IL-10 levels were higher (**Supplementary Figures S2L, M**, IL-1b: p = 0.019; IL-10: p = 0.022). Furthermore, the levels of IL-12 were lower (**Supplementary Figure S2N**, p = 0.020) in patients receiving chemoimmunotherapy as second or further lines treatment.

### 3.3 Survival analysis and cox regression of baseline serum cytokine levels in association with PFS

**Figure 1:**
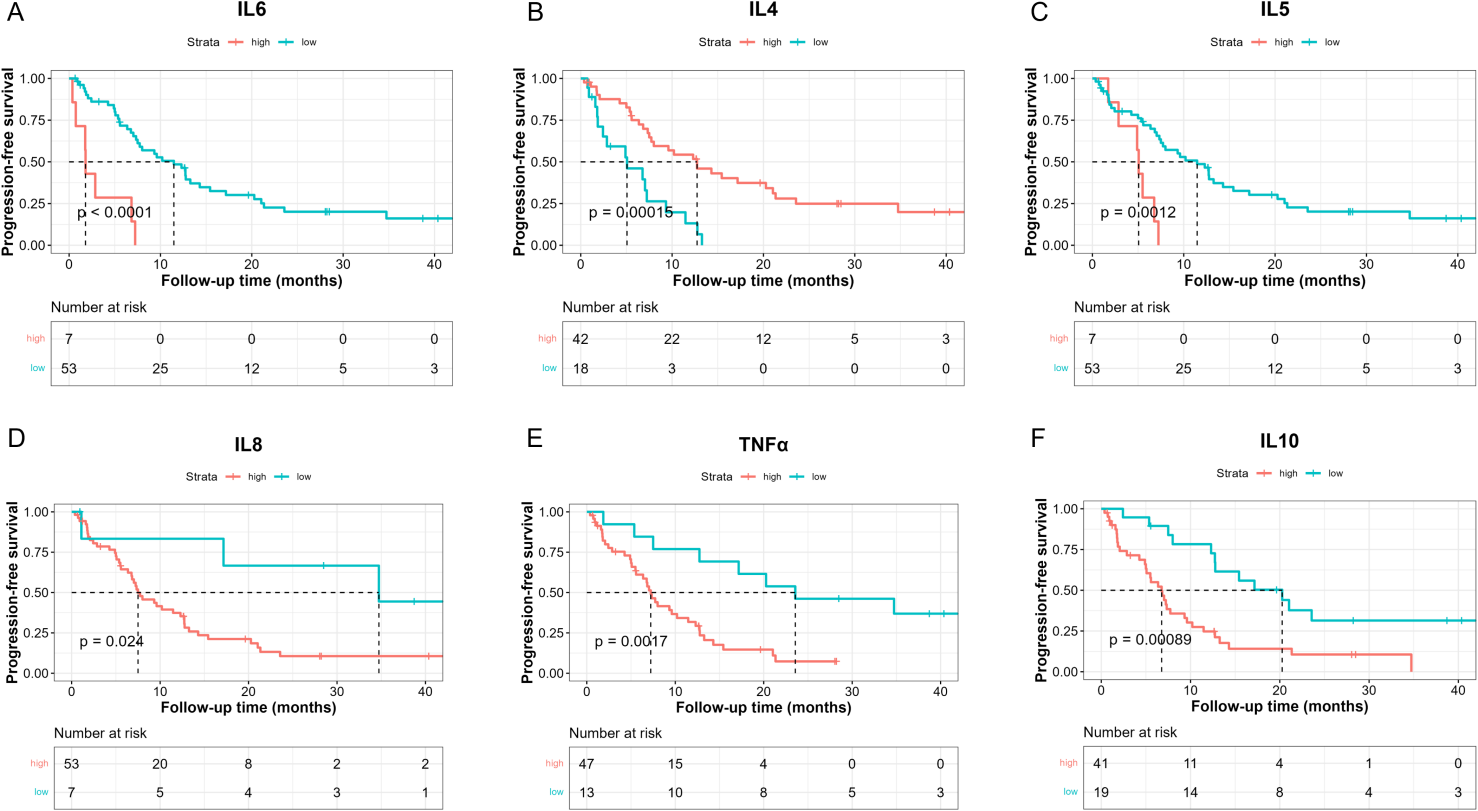
Kaplan-Meier analysis of PFS between high and low baseline serum IL-6 **(A)**, IL-4 **(B)**, IL-5 **(C)**, IL-8 **(D)**, TNF-α **(E)**, IL-10 **(F)** levels based on the determined cut-point.

We then investigated whether serum levels of the 10 cytokines could be potential prognostic factors for NSCLC patients treated with chemoimmunotherapy. The median PFS of 60 patients was 8 months (**Supplementary Figure S3**, 95%CI: [6.83, 12.8]). Then we determined the optimal cut-point for each cytokine and performed survival analysis in terms of PFS (**Table 2**). Our results suggested that patients with lower baseline IL-6 levels (< 24.05 pg/ml) had longer median PFS (**Figure 1A**, 11.5 [7.5, 15.4] vs 1.8 [0.733, NE], log-rank p = 0.000009). Patients with higher baseline IL-4 levels (> 0.1907 pg/ml) had better PFS (**Figure 1B**,12.73 [7.73, 21.3] vs 5.07 [2.43, 11.5], log-rank p = 0.00015). On the other hand, higher IL-5 levels (> 0.8236 pg/ml) were associated with poorer PFS (**Figure 1C**, 5.07 [2.87, NE] vs 11.47 [7.5, 15.4], log-rank p = 0.0012). Meanwhile, patients with lower levels IL-8 also had longer PFS (**Figure 1D**, 34.7 [17.17, NE] vs 7.5 [6.77, 12.7], log-rank p = 0.024). Higher TNF-α levels were related to poorer PFS (**Figure 1E**, 7.23 [5.57, 11.5] vs 23.57 [12.73, NE], log-rank p = 0.0017), and higher levels were related to poorer PFS as well (**Figure 1F**, 6.77 [5, 9.6] vs 20.27 [12.7, NE], log-rank p = 0.00089).

**Table 2:** Optimal cutpoint determination of patients’ baseline level of 10 cytokines and Kaplan-Meier analysis associated with PFS.

Cytokine	Cutpoint (pg/ml)	Level	N	mPFS (months with 95%CI)	Log-rank p
**IL-6**	**24.05**	**high**	**7**	**1.8 [0.733, NE]**	**0.000009**
		**low**	**53**	**11.5 [7.5, 15.4]**	
IL-12	0.2822	high	10	9.33 [7.23, NE]	0.2
		low	50	8 [6.37, 12.8]	
IL-1b	0.0799	high	27	10.2 [7.23, NE]	0.19
		low	33	8 [5.07, 13.3]	
**IL-4**	**0.1907**	**high**	**42**	**12.73 [7.73, 21.3]**	**0.00015**
		**low**	**18**	**5.07 [2.43, 11.5]**	
**IL-5**	**0.8236**	**high**	**7**	**5.07 [2.87, NE]**	**0.0012**
		**low**	**53**	**11.47 [7.5, 15.4]**	
IFN-γ	0.0065	high	54	7.73 [6.83, 12.7]	0.06
		low	6	21.33 [5, NE]	
**IL-8**	**3.776**	**high**	**53**	**7.5 [6.77, 12.7]**	**0.024**
		**low**	**7**	**34.7 [17.17, NE]**	
IL-22	2.905	high	9	6.77 [1.8, NE]	0.072
		low	51	9.6 [7.23, 14.3]	
**TNF-α**	**1.918**	**high**	**47**	**7.23 [5.57, 11.5]**	**0.0017**
		**low**	**13**	**23.57 [12.73, NE]**	
**IL-10**	**0.6092**	**high**	**41**	**6.77 [5, 9.6]**	**0.00089**
		**low**	**19**	**20.27 [12.7, NE]**	

Cytokines with statistical significance (Log-rank p < 0.05) are marked in bold.

To further investigate the role of baseline levels of these 10 cytokines in PFS, we utilized univariable and multivariable cox regression (**Table 3**). Results of univariable analysis showed that higher IL-6, IL-5, and IL-10 were associated with poorer PFS and were statistically significant. Given the number of events, we included two most statistically significant cytokines in the multivariable analysis. Cox regression results indicated that higher IL-6 and IL-5 were significantly associated with worse PFS (**Table 3**).

**Table 3:** Univariable and multivariable analysis of the baseline level of 10 cytokines in association with PFS.

Cytokine	Univariable			Multivariable		
	HR	95%CI	pvalue	HR	95%CI	p value
**IL-6**	**1.0536**	**1.05 [1.03, 1.08]**	**0.00004660**	**1.05284**	**1.05 [1.03, 1.08]**	**0.0000533**
IL-12	1.091094	1.09 [0.75, 1.58]	0.64277435			
IL-1b	2.039326	2.04 [0.56, 7.41]	0.27893968			
IL-4	1.039993	1.04 [0.84, 1.29]	0.72359181			
**IL-5**	**1.89344**	**1.89 [1.15, 3.13]**	**0.01278810**	**1.90155**	**1.90 [1.09, 3.32]**	**0.0235**
IFN-γ	1.666321	1.67 [0.82, 3.40]	0.16058774			
IL-8	1.011658	1.01 [0.99, 1.03]	0.24251668			
IL-22	1.253463	1.25 [0.99, 1.59]	0.06304361			
TNF-α	1.053082	1.05 [0.99, 1.12]	0.07865187			
**IL-10**	**1.373094**	**1.37 [1.03, 1.83]**	**0.02982810**			

Cytokines with statistical significance (p < 0.05) are marked in bold.

### 3.4 Survival analysis and cox regression of baseline serum cytokine levels in association with OS

**Figure 2:**
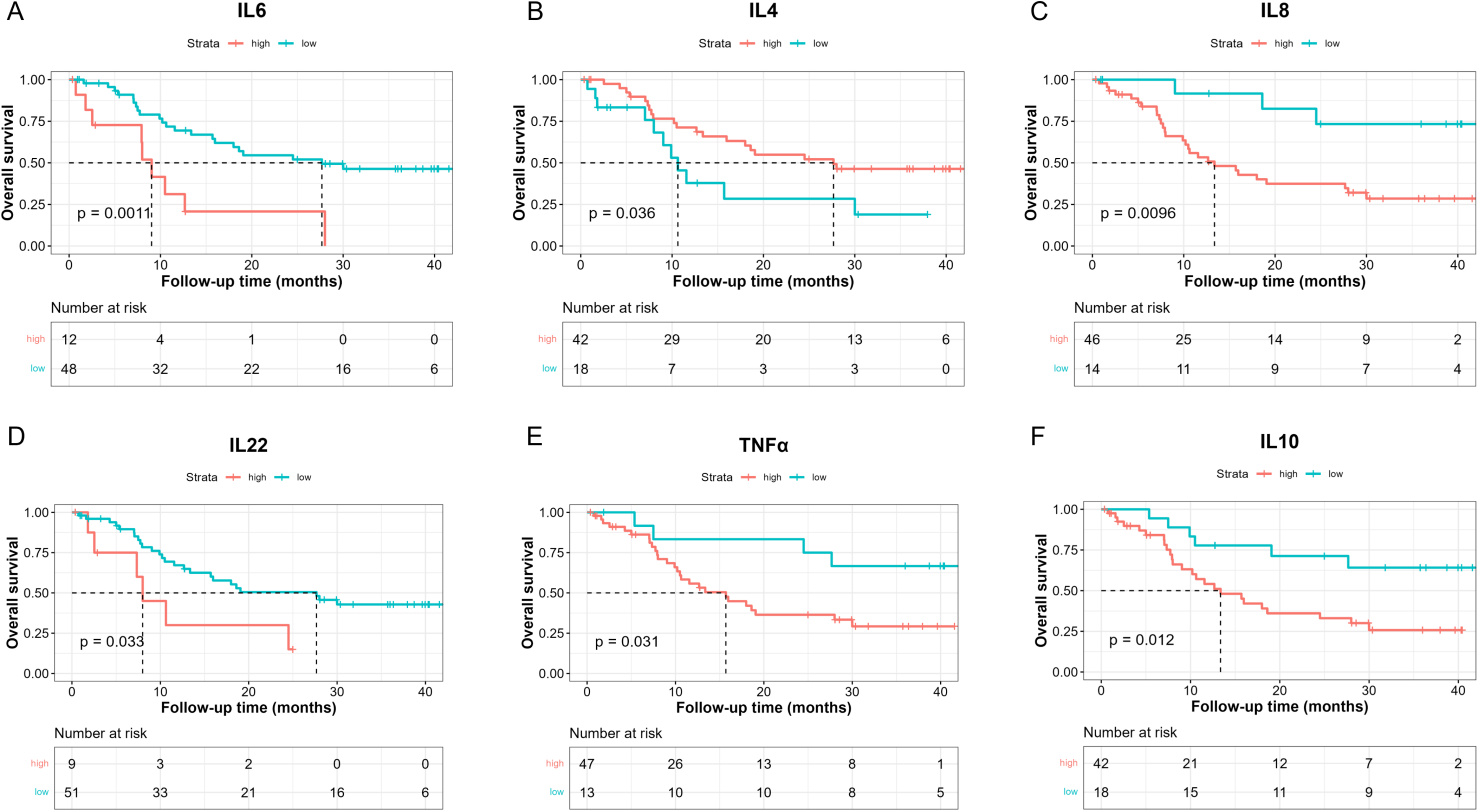
Kaplan-Meier analysis of OS between high and low baseline serum IL-6 **(A)**, IL-4 **(B)**, IL-8 **(C)**, IL-22 **(D)**, TNF-α **(E)**, IL-10 **(F)** levels based on the determined cut-point.

To investigate the prognostic effects of these 10 cytokines in terms of OS, we adopted the optimal cut-point determination method and survival analysis as well (**Table 4**). The median OS was 18.6 months in these patients (**Supplementary Figure S4**, 95%CI: [12.7, NE]). Results showed that lower IL-6 levels (< 18.67 pg/ml) were in relation with better OS (**Figure 2A**, 27.67 [15.97, NE] vs 9.03 [7.93, NE], log-rank p = 0.0011), while higher IL-4 levels (> 0.1907 pg/ml) suggested better OS (**Figure 2B**, 27.7 [16, NE] vs 10.6 [8, NE], log-rank p = 0.036). Moreover, patients with lower IL-8 levels (< 5.535 pg/ml) had longer OS (**Figure 2C**, NE [NE, NE] vs 13.4 [10.2, 30], log-rank p = 0.0096), and patients with lower IL-22 (< 2.905 pg/ml) also had longer OS (**Figure 2D**, 27.7 [13.37, NE] vs 8 [7.33, NE], log-rank p = 0.033). Lastly, higher TNF-α levels (> 1.918 pg/ml) indicated poorer OS (**Figure 2E**, 15.7 [10.5, 30] vs NE [27.7, NE], log-rank p = 0.031), and higher IL-10 levels (> 0.6034 pg/ml) indicated poorer OS as well (**Figure 2F**, 13.4 [9.03, 30] vs NE [27.67, NE], log-rank p = 0.012).

**Table 4:** Optimal cutpoint determination of patients’ baseline level of 10 cytokines and Kaplan-Meier analysis associated with OS.

Cytokine	Cutpoint (pg/ml)	Level	N	mOS (months with 95%CI)	Log-rank p
**IL-6**	**18.67**	**high**	**12**	**9.03 [7.93, NE]**	**0.0011**
		**low**	**48**	**27.67 [15.97, NE]**	
IL-12	0.2657	high	12	NE [15.7, NE]	0.24
		low	48	18 [10.6, NE]	
IL-1b	0.0226	high	54	16 [10.6, NE]	0.18
		low	6	NE [30, NE]	
**IL-4**	**0.1907**	**high**	**42**	**27.7 [16, NE]**	**0.036**
		**low**	**18**	**10.6 [8, NE]**	
IL-5	0.1332	high	38	12.7 [10.2, NE]	0.096
		low	22	NE [18.6, NE]	
IFN-γ	0.1672	high	15	15.7 [7.93, NE]	0.099
		low	45	27.7 [12.67, NE]	
**IL-8**	**5.535**	**high**	**46**	**13.4 [10.2, 30]**	**0.0096**
		**low**	**14**	**NE [NE, NE]**	
**IL-22**	**2.905**	**high**	**9**	**8 [7.33, NE]**	**0.033**
		**low**	**51**	**27.7 [13.37, NE]**	
**TNF-α**	**1.918**	**high**	**47**	**15.7 [10.5, 30]**	**0.031**
		**low**	**13**	**NE [27.7, NE]**	
**IL-10**	**0.6034**	**high**	**42**	**13.4 [9.03, 30]**	**0.012**
		**low**	**18**	**NE [27.67, NE]**	

Cytokines with statistical significance (Log-rank p < 0.05) are marked in bold.

Cox regression was also conducted to estimate the association between baseline cytokine levels and OS. Univariable analysis showed that higher baseline IL-6 levels were associated with worse OS (**Table 5**).

**Table 5:** Univariable analysis of the baseline level of 10 cytokines in association with OS.

Cytokine	Univariable		
	HR	95%CI	pvalue
**IL-6**	**1.054795**	**1.05 [1.02, 1.09]**	**0.000370**
IL-12	0.970001	0.97 [0.49, 1.93]	0.930703
IL-1b	0.777031	0.78 [0.05, 11.53]	0.854556
IL-4	0.92381	0.92 [0.57, 1.49]	0.744581
IL-5	1.233601	1.23 [0.66, 2.29]	0.507429
IFN-γ	1.098882	1.10 [0.42, 2.85]	0.846218
IL-8	1.017797	1.02 [0.99, 1.04]	0.141917
IL-22	1.247759	1.25 [0.91, 1.71]	0.170631
TNF-α	1.030217	1.03 [0.92, 1.16]	0.611796
IL-10	1.237002	1.24 [0.88, 1.74]	0.221491

CCytokines with statistical significance (p < 0.05) are marked in bold.

### 3.5 Time-dependent ROC analysis of baseline serum cytokine levels associated with PFS and OS

**Figure 3:**
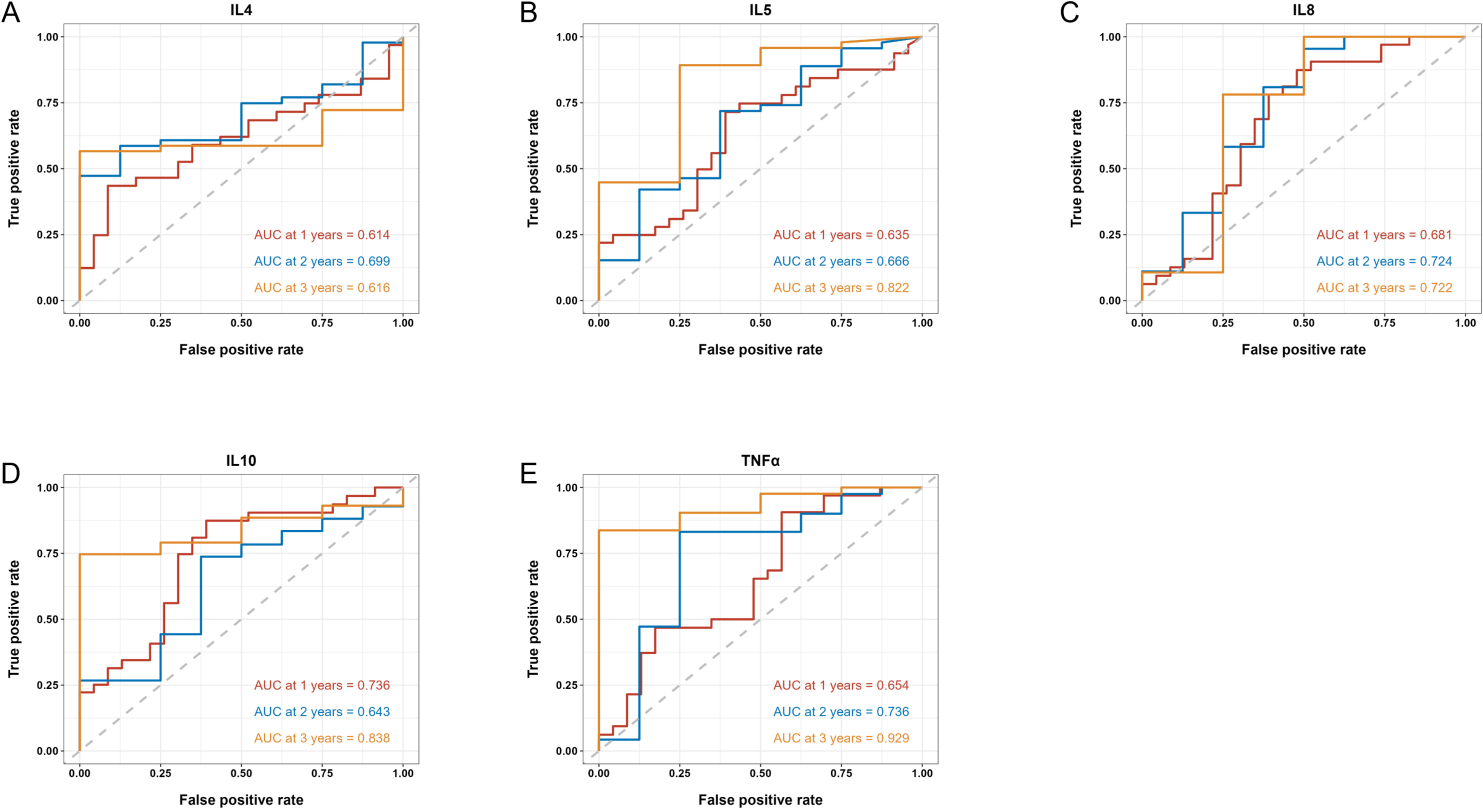
Time-dependent ROC of the baseline serum IL-4 **(A)**, IL-5 **(B)**, IL-8 **(C)**, IL-10 **(D)** and TNF-α **(E)** levels in association with PFS at 1, 2 and 3 years. AUC is shown separately.

**Figure 4:**
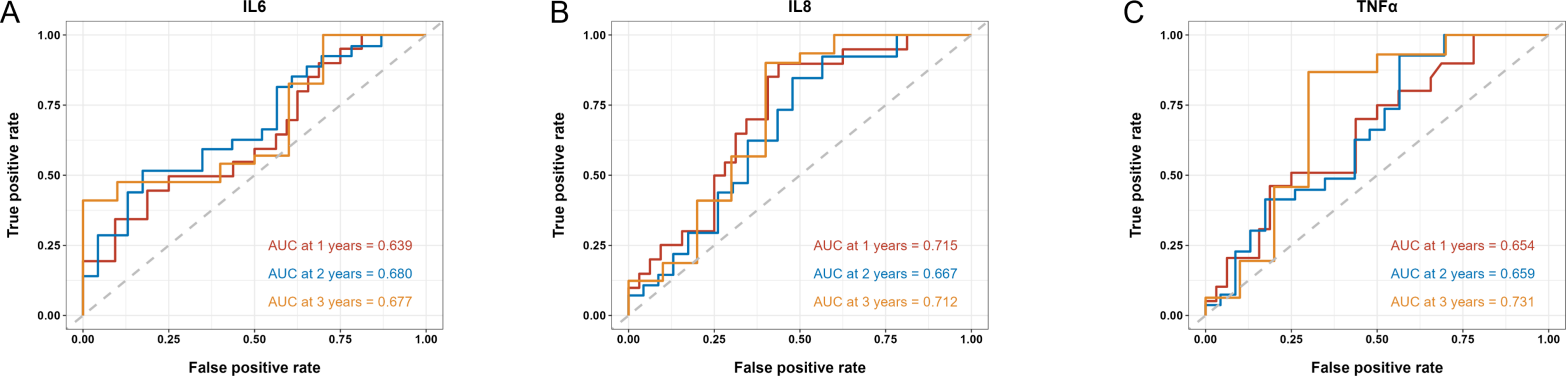
Time-dependent ROC of the baseline serum IL-6 **(A)**, IL-8 **(B)** and TNF-α **(C)** levels in association with OS at 1, 2 and 3 years. AUC is shown separately.

We then applied time-dependent ROC analysis to assess the sensitivity and specificity of serum cytokine levels (pg/ml) as prognostic factors in PFS and OS from another perspective. Cytokines with AUC > 0.6 at all three time points were considered acceptable prognostic factors. In terms of PFS, results indicated that IL-4, IL-5, IL-8, IL-10 and TNF-α levels had good predictive performance (**Figure 3**). IL-6, IL-8 and TNF-α levels were reasonable prognostic predictors for OS (**Figure 4**). We then examined whether the combination of serum cytokine levels could yield better prognostic effects. Combinations with AUC at two or three time points higher than singular results were considered better. As shown in **Supplementary Table S3**, **Supplementary Table S4**, IL-5 + TNF-α could serve as a prognostic indicator for PFS (AUC at 1 year = 0.657, AUC at 2 years = 0.740, AUC at 3 years = 0.936).

### 3.6 Changes of serum cytokine levels from baseline and its association with treatment response

During the study, we acquired dynamic serum samples from 19 patients at baseline and after the first assessment (two treatment cycles). Among these 19 patients, 5 patients had partial response (PR), 11 had stable disease (SD), and 3 had progressive disease (PD). First, we preliminarily investigated whether the serum levels of 10 cytokines were altered (**Supplementary Figure S5**). Results showed that levels of IL-10 and IFN-γ were significantly elevated after two cycles of treatment (IL-10: **Supplementary Figure S5E**, 0.6734 vs 1.16, p = 0.0012; IFN-γ: **Supplementary Figure S5I**, 0.0815 vs 0.1, p = 0.0053).

**Figure 5:**
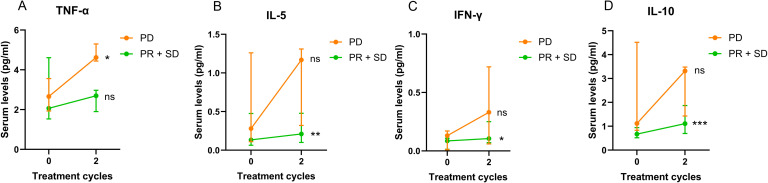
Changes of 19 patients’ serum TNF-α **(A)**, IL-5 **(B)**, IFN-γ **(C)** and IL-10 **(D)** levels after two treatment cycles in association with treatment response. Data are shown as median with lower and upper quartile. * p < 0.05; ** p < 0.01; *** p < 0.001; ns, not significant.

We then divided the 19 patients into two groups – PD and SD + PR, and serum cytokine levels were compared between these two groups. Results are presented in **Figure 5**; **Supplementary Table S5**. Our findings indicated that serum TNF-α levels were significantly increased in patients with PD after two cycles (p = 0.0185), while TNF-α levels in patients with SD or PR did not show significant changes (**Figure 5A**). Additionally, serum levels of IL-5, IFN-γ and IL-10 were significantly elevated in patients with SD or PR (IL-5: **Figure 5B**, p = 0.0076; IFN-γ: **Figure 5C**, p = 0.0335; IL-10: **Figure 5D**, p = 0.0003), but did not significantly change in patients with PD.

## 4 Discussion

In this study, we explored 10 serum cytokines as prognostic biomarkers in patients with NSCLC treated with chemotherapy combined with PD-1 inhibitor-based therapy.

Firstly, based on the determined optimal cut-point, our findings showed that patients with higher levels of baseline serum IL-6, IL-5, IL-8, TNF-α, IL-10 and low IL-4 levels had shorter PFS. Higher IL-6 and IL-5 levels were associated with poorer PFS. Secondly, higher levels of baseline serum IL-6, IL-8, IL-22, TNF-α, IL-10 or low levels of IL-4 were observed in patients with shorter OS, and higher IL-6 levels acted as an independent factor with worse OS. Lastly, we found that the increase of TNF-α levels was associated with worse treatment response, and increased IL-5, IFN-γ and IL-10 levels were associated with better treatment response.

IL-6 has been found to be deregulated in cancer, with its overexpression reported in almost all types of tumors (19). Our results indicated that higher baseline IL-6 levels were associated with shorter PFS and OS. A systematic analysis of IL-6 as a predictive biomarker in NSCLC patients also revealed that those with a low baseline concentration of IL-6 in serum specimens or tumor tissues could derive more benefit from immune checkpoint inhibitors (ICIs) (20). Similarly, in biliary tract cancers, high pretreatment levels of IL-6 and increasing levels during treatment were associated with short PFS and OS (21).

Moreover, we found that patients with lymph node metastasis had higher baseline TNF-α levels and they were related to poorer PFS as well. However, a previous study found that an increase in TNF-α levels measured with flow cytometry suggested a better response to ICIs in NSCLC (22). While aligning with our results, another study showed that elevated TNF-α could indicate a worse treatment response (23). The conflict might be due to different sensitivity of detecting methods and clinical background, thus further studies are needed.

IL-8 is a pro-inflammatory cytokine and can promote angiogenesis (24). Published studies showed elevated levels of IL-8 were associated with poor prognosis of ICIs treatment in several types of cancer, including NSCLC (24–26). Similarly, our results suggested that patients with higher baseline levels of IL-8 had shorter PFS and OS, and a decrease of IL-8 levels could indicate better response to chemoimmunotherapy.

Furthermore, IL-5 and IL-10 also play significant roles in the immune response. A study indicated that patients with a low baseline concentration of IL-5 in serum specimens or tumor tissues could derive more benefit from ICIs (27), which was similar to our findings. On the other hand, high expression of serous IL–10 could lead to an adverse survival in most types of cancer and was related to the risk of immune-related adverse events (28, 29).

IL-4 is reported as one of immune-suppressive cytokines in tumor microenvironment that help tumor growth and metastasis (30). However, the role of IL-4 as a biomarker for immunotherapy-based treatments remains to be clarified. Our study found that patients with brain metastasis had lower baseline IL-4 levels, and patients bearing KRAS mutations had higher IL-4 levels. Patients with brain metastasis are often considered having poorer prognosis, while bearing KRAS mutations is a possible positive prognostic indicator for immunotherapy response based on previous studies (31). In alignment with these findings, our results demonstrated that higher baseline levels of IL-4 were associated with both better PFS and better OS.

The evidence above supports the findings that various serum cytokine could act as prognostic biomarkers in NSCLC treated with PD-1 inhibitor combined with chemotherapy. However, there were several limitations in this study. Firstly, it was a single-center study conducted in a Chinese population with a small sample size, which may lead to statistical biases and limit its interpretation and broader application. Secondly, patients received different chemotherapy and immunotherapy reagents in different treatment lines, and subgroup analysis was not feasible due to the small sample size as well.

In conclusion, our study suggests that several serum cytokine levels, including IL-6, IL-5, IL-8, TNF-α, IL-10, and IL-4, could serve as prognostic indicators in NSCLC patients receiving chemotherapy and PD-1 inhibitor-based treatment.

Higher IL-6 and IL-5 indicated worse survivals independently. Increase of serum TNF-α levels from baseline was associated with worse treatment response, while increase of serum IL-5, IFN-γ and IL-10 levels was associated with better treatment response. These findings could potentially guide treatment decisions and improve patient outcomes. However, further validation through multi-center studies with larger sample sizes is needed.

## Data Availability

The raw data supporting the conclusions of this article will be made available by the authors, without undue reservation.

## Supplementary material

Supplementary Figure 1 Boxplot of serum baseline cytokines among different treatment response categories.

Supplementary Figure 2 Boxplot of serum baseline cytokines among different clinicopathological groups with statistical significance.

Supplementary Figure 3 Kaplan-Meier analysis of overall PFS of 60 patients receiving chemoimmunotherapy.

Supplementary Figure 4 Kaplan-Meier analysis of overall OS of 60 patients receiving chemoimmunotherapy.

Supplementary Figure 5 Changes of 19 patients’ serum cytokine levels after 2 treatment cycles (red: PD, green: PR, dark cyan: SD).
