# Supplementary material for: Prognostic role of serum cytokines level in non-small cell lung cancer patients with anti-PD-1 and chemotherapy combined treatment
Source: Front Immunol. 2024 Oct 22;15:1430301. doi: 10.3389/fimmu.2024.1430301 (PMC11534701; doi:10.3389/fimmu.2024.1430301)
Supplement: Supplementary file 1 [file Table1.docx]

Table S1. The association of patients’ baseline clinicopathological characteristics and PFS or OS.

| **Characteristic** |  |  | **N** | **%** | **PFS** | | | **OS** | | |
| --- | --- | --- | --- | --- | --- | --- | --- | --- | --- | --- |
|  |  |  |  |  | **HR** | **95%CI** | **pvalue** | **HR** | **95%CI** | **pvalue** |
| Age |  | ≤60 | 13 | 21.67% | 1.231185 | 1.23 [0.61, 2.49] | 0.562695 | 1.085557 | 1.09 [0.47, 2.53] | 0.848857 |
|  |  | >60 | 47 | 78.33% |  |  |  |  |  |  |
| Sex |  | Male | 52 | 86.67% | 0.625095 | 0.63 [0.28, 1.41] | 0.257914 | 0.652352 | 0.65 [0.23, 1.88] | 0.428182 |
|  |  | Female | 8 | 13.33% |  |  |  |  |  |  |
| Histology |  | Adenocarcinoma | 30 | 50.00% | 0.868616 | 0.87 [0.47, 1.59] | 0.649455 | 0.771184 | 0.77 [0.37, 1.62] | 0.491599 |
|  |  | Squamous cell carcinoma | 25 | 41.67% |  |  |  |  |  |  |
|  |  | Non-small cell lung cancer | 5 | 8.33% |  |  |  |  |  |  |
| TNM system | T | 1 | 7 | 11.67% |  |  |  |  |  |  |
|  |  | 2 | 18 | 30.00% | 1.006915 | 1.01 [0.39, 2.59] | 0.988586 | 0.710123 | 0.71 [0.21, 2.36] | 0.576469 |
|  |  | 3 | 4 | 6.67% | 0.707015 | 0.71 [0.17, 2.86] | 0.627042 | 1.800753 | 1.80 [0.40, 8.11] | 0.443729 |
|  |  | 4 | 31 | 51.67% | 0.594589 | 0.59 [0.23, 1.51] | 0.275398 | 0.867143 | 0.87 [0.29, 2.60] | 0.799081 |
|  | N | 0 | 7 | 11.67% |  |  |  |  |  |  |
|  |  | 1 | 0 | 0.00% |  |  |  |  |  |  |
|  |  | 2 | 27 | 45.00% | 0.740609 | 0.74 [0.32, 1.73] | 0.488955 | 0.766536 | 0.77 [0.28, 2.13] | 0.610233 |
|  |  | 3 | 25 | 41.67% | 0.566971 | 0.57 [0.23, 1.40] | 0.21887 | 0.635703 | 0.64 [0.22, 1.83] | 0.401939 |
|  |  | X | 1 | 1.67% |  |  |  |  |  |  |
|  | M | 0 | 22 | 36.67% |  |  |  |  |  |  |
|  |  | 1 | 38 | 63.33% | 1.32689 | 1.33 [0.71, 2.47] | 0.370768 | **2.678017** | **2.68 [1.15, 6.26]** | **0.022875** |
|  | Stage | I | 2 | 3.33% | 0.998929 | 1.00 [0.59, 1.68] | 0.996783 | **2.587880** | **2.59 [1.15, 5.84]** | **0.021923** |
|  |  | II | 0 | 0.00% |  |  |  |  |  |  |
|  |  | III | 20 | 33.33% |  |  |  |  |  |  |
|  |  | IV | 38 | 63.33% |  |  |  |  |  |  |
| Bone Metastasis |  | No | 45 | 75.00% |  |  |  |  |  |  |
|  |  | Yes | 15 | 25.00% | 1.403130 | 1.40 [0.74, 2.66] | 0.298209 | 1.563133 | 1.56 [0.75, 3.28] | 0.237188 |
| Brain Metastasis |  | No | 55 | 91.67% |  |  |  |  |  |  |
|  |  | Yes | 5 | 8.33% | 0.960715 | 0.96 [0.34, 2.69] | 0.939196 | 0.390301 | 0.39 [0.05, 2.87] | 0.354950 |
| Lung Metastasis |  | No | 48 | 80% |  |  |  |  |  |  |
|  |  | Yes | 12 | 20% | 1.197376 | 1.20 [0.59, 2.42] | 0.615593 | 1.671271 | 1.67 [0.72, 3.90] | 0.235562 |
| Pleural Metastasis |  | No | 53 | 88.33% |  |  |  |  |  |  |
|  |  | Yes | 7 | 11.67% | 0.933411 | 0.93 [0.33, 2.62] | 0.895794 | 2.014789 | 2.01 [0.77, 5.27] | 0.153499 |
| Lymph node Metastasis |  | No | 55 | 91.67% |  |  |  |  |  |  |
|  |  | Yes | 5 | 8.33% | **4.104974** | **4.10 [1.51, 11.15]** | **0.005620** | **5.120611** | **5.12 [1.58, 16.63]** | **0.006564** |
| Mutation status | KRAS | No | 34 | 56.67% |  |  |  |  |  | 0.531627 |
|  |  | Yes | 13 | 21.67% | 1.127359 | 1.13 [0.54, 2.34] | 0.748348 | 1.312361 | 1.31 [0.56, 3.08] |  |
|  |  | Undetermined | 13 | 21.67% |  |  |  |  |  |  |
|  | EGFR | No | 51 | 85.00% |  |  |  |  |  | 0.100256 |
|  |  | Yes | 3 | 5.00% | 1.572623 | 1.57 [0.48, 5.17] | 0.455626 | 2.751725 | 2.75 [0.82, 9.20] |  |
|  |  | Undetermined | 6 | 10.00% |  |  |  |  |  |  |
|  | TP53 | No | 5 | 8.33% |  |  |  |  |  | 0.387060 |
|  |  | Yes | 26 | 43.33% | 1.305568 | 1.31 [0.44, 3.86] | 0.629921 | 1.921910 | 1.92 [0.44, 8.45] |  |
|  |  | Undetermined | 29 | 48.33% |  |  |  |  |  |  |
| PD-L1 expression |  | <1% | 12 | 20.00% |  |  |  |  |  |  |
|  |  | 1%~50% | 11 | 18.33% | 0.789151 | 0.79 [0.32, 1.92] | 0.601425 | 0.616546 | 0.62 [0.24, 1.57] | 0.31085 |
|  |  | ≥50% | 18 | 30.00% | **0.356057** | **0.36 [0.15, 0.85]** | **0.020216** | **0.192347** | **0.19 [0.06, 0.58]** | **0.003185** |
|  |  | Undetermined | 19 | 31.67% |  |  |  |  |  |  |
| Treatment-line |  | 1 | 43 | 71.67% |  |  |  |  |  |  |
|  |  | ≥2 | 17 | 28.33% | 1.518126 | 1.52 [0.78, 2.96] | 0.219472 | 1.515193 | 1.52 [0.70, 3.30] | 0.295272 |
| Treatment response |  | PD | 13 | 21.67% |  |  |  |  |  |  |
|  |  | SD | 31 | 51.67% | **0.063280** | **0.06 [0.02, 0.17]** | **2.97E-08** | 0.522241 | 0.52 [0.24, 1.16] | 0.109524 |
|  |  | PR | 16 | 26.67% | **0.017088** | **0.02 [0.01, 0.06]** | **5.29E-11** | **0.033521** | **0.03 [0.00, 0.27]** | **0.001384** |
|  |  | CR | 0 | 0.00% |  |  |  |  |  |  |

Table S2. The levels of 10 cytokines in different clinicopathological subgroups and Mann-Whitney U test analysis

| **Characteristic** |  |  | **IL-6 (pg/ml)** | | | **IL-12 (pg/ml)** | | | **IL-1b (pg/ml)** | | | **IL-4 (pg/ml)** | | | **IL-5 (pg/ml)** | | | **IFN-γ (pg/ml)** | | | **IL-8 (pg/ml)** | | | **IL-22 (pg/ml)** | | |  | | | **TNF-α (pg/ml)** | | | **IL-10 (pg/ml)** | | |
| --- | --- | --- | --- | --- | --- | --- | --- | --- | --- | --- | --- | --- | --- | --- | --- | --- | --- | --- | --- | --- | --- | --- | --- | --- | --- | --- | --- | --- | --- | --- | --- | --- | --- | --- | --- |
|  |  |  | **Q1** | **Median** | **Q3** | **Q1** | **Median** | **Q3** | **Q1** | **Median** | **Q3** | **Q1** | **Median** | **Q3** | **Q1** | **Median** | **Q3** | **Q1** | **Median** | **Q3** | **Q1** | **Median** | **Q3** | **Q1** | **Median** | **Q3** |  | | | **Q1** | **Median** | **Q3** | **Q1** | **Median** | **Q3** |
| **Age** |  | **≤60** | 1.492 | 2.970 | 8.716 | 0.080 | 0.142 | 0.225 | 0.026 | 0.052 | 0.099 | 0.162 | 0.593 | 1.084 | 0.042 | **0.086** | 0.161 | 0.000 | 0.062 | 0.163 | 5.536 | 10.040 | 17.490 | 0.463 | 0.868 | 2.243 |  | | | 1.833 | 2.244 | 2.789 | 0.508 | 0.722 | 0.986 |
|  |  | **>60** | 3.807 | 6.808 | 18.670 | 0.068 | 0.112 | 0.262 | 0.038 | 0.080 | 0.145 | 0.161 | 0.358 | 0.610 | 0.133 | **0.256** | 0.638 | 0.047 | 0.101 | 0.244 | 6.469 | 9.819 | 17.650 | 1.032 | 1.453 | 2.709 |  | | | 1.962 | 2.758 | 4.583 | 0.544 | 0.927 | 1.332 |
|  |  | **p value** |  |  |  |  |  |  |  |  |  |  |  |  |  | **0.001** |  |  |  |  |  |  |  |  |  |  |  | | |  |  |  |  |  |  |
| **Sex** |  | **Male** | 3.103 | 7.425 | 17.875 | 0.084 | 0.126 | 0.241 | 0.037 | 0.080 | 0.144 | 0.195 | 0.397 | 0.747 | 0.116 | 0.209 | 0.611 | 0.043 | 0.106 | 0.233 | 5.589 | 10.510 | 17.920 | 0.973 | 1.408 | 2.733 |  | | | 1.965 | 2.718 | 4.338 | 0.541 | 0.806 | 1.263 |
|  |  | **Female** | 1.377 | 3.063 | 6.312 | 0.026 | 0.103 | 0.269 | 0.025 | 0.036 | 0.073 | 0.127 | 0.160 | 0.992 | 0.022 | 0.080 | 0.246 | 0.005 | 0.045 | 0.098 | 7.184 | 9.753 | 13.823 | 0.317 | 0.422 | 1.486 |  | | | 1.598 | 2.167 | 3.019 | 0.628 | 0.923 | 1.286 |
|  |  | **p value** | 0.037 |  |  |  |  |  |  |  |  |  |  |  |  |  |  | 0.043 |  |  |  |  |  | 0.017 |  |  |  | | |  |  |  |  |  |  |
| **Histology** |  | **Adenocarcinoma** | 1.765 | 4.891 | 10.992 | 0.032 | **0.103** | 0.254 | 0.028 | **0.059** | 0.099 | 0.161 | 0.471 | 0.782 | 0.085 | 0.196 | 0.408 | 0.020 | **0.077** | 0.121 | 6.750 | 10.355 | 15.910 | 0.690 | 1.358 | 2.377 |  | | | 1.928 | 2.379 | 3.152 | 0.602 | 0.857 | 1.290 |
|  |  | **Squamous cell carcinoma** | 2.720 | 5.268 | 12.620 | 0.105 | **0.147** | 0.310 | 0.043 | **0.092** | 0.173 | 0.144 | 0.338 | 0.684 | 0.127 | 0.395 | 0.849 | 0.061 | **0.121** | 0.300 | 5.157 | 10.980 | 21.515 | 0.978 | 1.453 | 3.119 |  | | | 1.978 | 2.888 | 4.443 | 0.460 | 0.780 | 1.332 |
|  |  | **p value** |  |  |  |  | **0.022** |  |  | **0.046** |  |  |  |  |  |  |  |  | **0.026** |  |  |  |  |  |  |  |  | | |  |  |  |  |  |  |
| **TNM system** | **T** | **1** | 1.417 | 1.774 | 6.808 | 0.018 | 0.025 | 0.141 | 0.025 | 0.032 | 0.050 | 0.115 | **0.130** | 0.383 | 0.089 | 0.191 | 0.441 | 0.026 | 0.095 | 0.244 | 6.670 | 6.764 | 11.240 | 0.342 | 1.581 | 2.807 |  | | | 1.928 | 2.771 | 3.131 | 0.671 | 1.089 | 1.332 |
|  |  | **2** | 3.598 | 4.758 | 16.285 | 0.093 | 0.136 | 0.231 | 0.036 | 0.059 | 0.148 | 0.156 | **0.270** | 0.573 | 0.123 | 0.459 | 0.770 | 0.086 | 0.119 | 0.393 | 3.882 | 7.731 | 12.078 | 1.036 | 1.285 | 2.983 |  | | | 2.311 | 3.028 | 4.797 | 0.530 | 0.872 | 1.310 |
|  |  | **3** | 1.952 | 2.910 | 7.442 | 0.101 | 0.123 | 0.234 | 0.030 | 0.066 | 0.127 | 0.461 | **1.600** | 2.545 | 0.044 | 0.200 | 0.264 | 0.003 | 0.016 | 0.379 | 4.692 | 12.154 | 28.983 | 0.377 | 1.175 | 3.385 |  | | | 1.579 | 1.963 | 4.529 | 0.360 | 0.698 | 0.852 |
|  |  | **4** | 4.831 | 8.078 | 21.130 | 0.071 | 0.126 | 0.282 | 0.042 | 0.085 | 0.148 | 0.210 | **0.440** | 0.938 | 0.073 | 0.187 | 0.542 | 0.027 | 0.072 | 0.132 | 8.099 | 13.620 | 21.170 | 0.868 | 1.363 | 2.476 |  | | | 1.738 | 2.230 | 3.737 | 0.513 | 0.831 | 1.285 |
|  |  | **p value** |  |  |  |  |  |  |  |  |  |  | **0.034** |  |  |  |  |  |  |  |  |  |  |  |  |  |  | | |  |  |  |  |  |  |
|  | **N** | **0** | 0.995 | 2.408 | 6.808 | 0.023 | **0.030** | 0.144 | 0.028 | 0.050 | 0.104 | 0.130 | 0.383 | 0.561 | 0.038 | 0.191 | 0.638 | 0.027 | 0.089 | 0.110 | 6.745 | 10.040 | 14.800 | 0.763 | 1.667 | 2.903 |  | | | 1.262 | 1.928 | 2.324 | 0.355 | 0.609 | 0.927 |
|  |  | **2** | 2.469 | 5.638 | 13.690 | 0.100 | **0.169** | 0.266 | 0.032 | 0.075 | 0.138 | 0.161 | 0.334 | 0.715 | 0.108 | 0.194 | 0.542 | 0.018 | 0.082 | 0.167 | 4.914 | 9.511 | 20.180 | 0.963 | 1.222 | 2.709 |  | | | 1.974 | 2.758 | 4.350 | 0.513 | 0.728 | 1.379 |
|  |  | **3** | 3.941 | 7.770 | 20.140 | 0.088 | **0.115** | 0.245 | 0.041 | 0.089 | 0.149 | 0.166 | 0.529 | 0.798 | 0.109 | 0.216 | 0.603 | 0.058 | 0.105 | 0.415 | 6.150 | 10.980 | 16.465 | 0.812 | 1.353 | 2.366 |  | | | 1.860 | 2.677 | 4.865 | 0.608 | 0.964 | 1.281 |
|  |  | **p value** |  |  |  |  | **0.022** |  |  |  |  |  |  |  |  |  |  |  |  |  |  |  |  |  |  |  |  | | |  |  |  |  |  |  |
|  |  | **0** | 2.197 | 4.989 | 13.815 | 0.083 | 0.132 | 0.233 | 0.032 | 0.065 | 0.116 | 0.180 | 0.347 | 0.647 | 0.123 | 0.413 | 0.836 | 0.053 | 0.108 | 0.347 | 4.334 | 8.300 | 14.628 | 0.921 | 1.187 | 2.734 |  | | | 2.112 | 2.887 | 4.372 | 0.510 | 0.762 | 1.215 |
|  | **M** | **1** | 2.454 | 6.500 | 16.285 | 0.065 | 0.120 | 0.254 | 0.037 | 0.078 | 0.143 | 0.161 | 0.397 | 0.809 | 0.092 | 0.189 | 0.419 | 0.025 | 0.089 | 0.135 | 6.391 | 11.065 | 18.085 | 0.844 | 1.447 | 2.542 |  | | | 1.926 | 2.308 | 3.758 | 0.584 | 0.905 | 1.344 |
|  |  | **p value** |  |  |  |  |  |  |  |  |  |  |  |  |  |  |  |  |  |  |  |  |  |  |  |  |  | | |  |  |  |  |  |  |
|  | **Stage** | **I** | 0.991 | 3.900 |  | 0.027 | 0.028 |  | 0.008 | 0.038 |  | 0.130 | 0.346 |  | 0.397 | 0.668 |  | 0.039 | 0.064 |  | 6.745 | 6.749 |  | 1.667 | 2.237 |  |  | | | 2.090 | 2.207 |  | 0.699 | 0.924 |  |
|  |  | **III** | 2.758 | 4.989 | 14.065 | 0.100 | 0.140 | 0.266 | 0.033 | 0.065 | 0.137 | 0.195 | 0.347 | 0.721 | 0.117 | 0.355 | 0.808 | 0.059 | 0.126 | 0.363 | 4.039 | 9.505 | 16.643 | 0.915 | 1.156 | 2.437 |  | | | 2.150 | 3.028 | 4.513 | 0.503 | 0.762 | 1.221 |
|  |  | **IV** | 2.454 | 6.500 | 16.285 | 0.065 | 0.120 | 0.254 | 0.037 | 0.078 | 0.143 | 0.161 | 0.397 | 0.809 | 0.092 | 0.189 | 0.419 | 0.025 | 0.089 | 0.135 | 6.391 | 11.065 | 18.085 | 0.844 | 1.447 | 2.542 |  | | | 1.926 | 2.308 | 3.758 | 0.584 | 0.905 | 1.344 |
|  |  | **p value** |  |  |  |  |  |  |  |  |  |  |  |  |  |  |  |  |  |  |  |  |  |  |  |  |  | | |  |  |  |  |  |  |
| **Bone Metastasis** | | **No** | 2.364 | 4.906 | 10.478 | 0.075 | 0.126 | 0.250 | 0.032 | 0.066 | 0.143 | 0.154 | 0.362 | 0.660 | 0.085 | 0.216 | 0.612 | 0.024 | 0.083 | 0.150 | 5.467 | 9.687 | 16.465 | 0.746 | **1.084** | 2.330 |  | | | 1.833 | 2.412 | 3.959 | 0.570 | 0.831 | 1.281 |
|  |  | **Yes** | 2.970 | 8.756 | 21.130 | 0.046 | 0.110 | 0.266 | 0.042 | 0.080 | 0.138 | 0.210 | 0.383 | 1.610 | 0.098 | 0.187 | 0.314 | 0.105 | 0.118 | 0.201 | 6.469 | 11.240 | 18.010 | 1.547 | **1.885** | 3.527 |  | | | 1.981 | 2.771 | 5.003 | 0.418 | 0.927 | 1.178 |
|  |  | **p value** |  |  |  |  |  |  |  |  |  |  |  |  |  |  |  |  |  |  |  |  |  |  | **0.008** |  |  | | |  |  |  |  |  |  |
| **Brain Metastasis** | | **No** | 2.970 | 6.808 | 14.190 | 0.071 | 0.126 | 0.251 | 0.034 | 0.067 | 0.138 | 0.191 | **0.411** | 0.766 | 0.096 | 0.199 | 0.619 | 0.039 | 0.101 | 0.201 | 6.469 | 10.040 | 18.010 | 0.896 | 1.353 | 2.709 |  | | | 1.962 | 2.677 | 4.302 | 0.597 | 0.831 | 1.224 |
|  |  | **Yes** | 0.486 | 1.834 | 44.323 | 0.041 | 0.115 | 0.243 | 0.031 | 0.113 | 0.230 | 0.098 | **0.140** | 0.286 | 0.064 | 0.209 | 0.236 | 0.010 | 0.047 | 0.109 | 3.615 | 8.481 | 13.050 | 0.652 | 1.064 | 2.189 |  | | | 0.855 | 2.270 | 3.538 | 0.542 | 0.973 | 1.922 |
|  |  | **p value** |  |  |  |  |  |  |  |  |  |  | **0.025** |  |  |  |  |  |  |  |  |  |  |  |  |  |  | | |  |  |  |  |  |  |
| **Lung Metastasis** |  | **No** | 1.830 | 7.085 | 15.165 | 0.064 | 0.113 | 0.209 | 0.035 | 0.066 | 0.111 | 0.142 | 0.347 | 0.811 | 0.090 | 0.189 | 0.578 | 0.036 | 0.092 | 0.165 | 5.691 | 9.753 | 17.920 | 0.879 | 1.285 | 2.791 |  | | | 1.940 | 2.546 | 4.069 | 0.519 | 0.857 | 1.221 |
|  |  | **Yes** | 3.769 | 5.072 | 11.233 | 0.078 | 0.213 | 0.296 | 0.032 | 0.080 | 0.302 | 0.248 | 0.511 | 0.713 | 0.181 | 0.212 | 0.587 | 0.032 | 0.108 | 0.957 | 6.877 | 11.100 | 15.115 | 0.932 | 1.447 | 2.193 |  | | | 1.794 | 2.545 | 4.635 | 0.605 | 0.798 | 2.377 |
|  |  | **p value** |  |  |  |  |  |  |  |  |  |  |  |  |  |  |  |  |  |  |  |  |  |  |  |  |  | | |  |  |  |  |  |  |
| **Pleural Metastasis** |  | **No** | 2.364 | 5.072 | 13.940 | 0.070 | 0.126 | 0.250 | 0.037 | 0.067 | 0.142 | 0.166 | 0.383 | 0.762 | 0.106 | 0.209 | 0.586 | 0.044 | **0.107** | 0.223 | 6.313 | 10.040 | 16.465 | 0.944 | 1.453 | 2.758 |  | | | 1.982 | 2.758 | 4.326 | 0.572 | 0.869 | 1.218 |
|  |  | **Yes** | 4.875 | 9.493 | 15.490 | 0.042 | 0.095 | 0.262 | 0.023 | 0.089 | 0.142 | 0.098 | 0.328 | 0.547 | 0.038 | 0.108 | 0.586 | 0.022 | **0.027** | 0.063 | 5.535 | 9.157 | 21.330 | 0.463 | 0.763 | 1.363 |  | | | 1.537 | 1.928 | 2.106 | 0.355 | 0.728 | 1.607 |
|  |  | **p value** |  |  |  |  |  |  |  |  |  |  |  |  |  |  |  |  | **0.026** |  |  |  |  |  |  |  |  | | |  |  |  |  |  |  |
| **Lymph node Metastasis** |  | **No** | 2.319 | 5.638 | 13.690 | 0.063 | 0.126 | 0.251 | 0.034 | 0.074 | 0.138 | 0.159 | 0.383 | 0.758 | 0.093 | 0.194 | 0.542 | 0.027 | 0.091 | 0.167 | 5.535 | 9.511 | 17.650 | 0.868 | 1.222 | 2.709 |  | | | 1.928 | **2.345** | 3.737 | 0.540 | 0.744 | 1.224 |
|  |  | **Yes** | 3.138 | 5.268 | 31.745 | 0.070 | 0.110 | 3.063 | 0.036 | 0.053 | 1.082 | 0.166 | 0.210 | 5.934 | 0.245 | 0.619 | 1.820 | 0.081 | 0.144 | 1.234 | 10.565 | 12.020 | 20.000 | 1.219 | 1.547 | 3.002 |  | | | 3.360 | **5.390** | 18.748 | 0.986 | 1.070 | 2.628 |
|  |  | **p value** |  |  |  |  |  |  |  |  |  |  |  |  |  |  |  |  |  |  |  |  |  |  |  |  |  | | |  | **0.003** |  |  |  |  |
| **Mutation status** | **KRAS** | **No** | 3.731 | 7.085 | 20.380 | 0.067 | 0.108 | 0.213 | 0.040 | 0.066 | 0.108 | 0.140 | **0.336** | 0.824 | 0.120 | 0.200 | 0.630 | 0.046 | 0.107 | 0.245 | 6.732 | 10.530 | 17.740 | 0.994 | 1.403 | 2.758 |  | | | 2.063 | 2.759 | 4.281 | 0.586 | 0.968 | 1.278 |
|  |  | **Yes** | 1.120 | 5.638 | 10.722 | 0.091 | 0.141 | 0.285 | 0.032 | 0.085 | 0.143 | 0.493 | **0.709** | 0.884 | 0.084 | 0.209 | 0.466 | 0.011 | 0.083 | 0.196 | 5.273 | 10.040 | 16.985 | 0.577 | 1.041 | 1.836 |  | | | 1.833 | 2.412 | 4.821 | 0.608 | 0.869 | 1.246 |
|  |  | **p value** |  |  |  |  |  |  |  |  |  |  | **0.041** |  |  |  |  |  |  |  |  |  |  |  |  |  |  | | |  |  |  |  |  |  |
|  | **EGFR** | **No** | 2.469 | 6.808 | 15.490 | 0.082 | 0.126 | 0.266 | 0.034 | 0.074 | 0.138 | 0.161 | 0.383 | 0.758 | 0.114 | **0.209** | 0.553 | 0.035 | 0.093 | 0.168 | 6.670 | 10.890 | 17.650 | 0.896 | 1.222 | 2.476 |  | | | 1.962 | 2.662 | 4.583 | 0.597 | 0.895 | 1.332 |
|  |  | **Yes** | 1.739 | 2.319 |  | 0.018 | 0.068 |  | 0.019 | 0.027 |  | 0.115 | 0.162 |  | 0.000 | **0.089** |  | 0.000 | 0.101 |  | 6.157 | 9.687 |  | 0.205 | 1.353 |  |  | | | 2.244 | 2.817 |  | 0.722 | 0.964 |  |
|  |  | **p value** |  |  |  |  |  |  |  |  |  |  |  |  |  | **0.033** |  |  |  |  |  |  |  |  |  |  |  | | |  |  |  |  |  |  |
|  | **TP53** | **No** | 2.476 | 4.875 | 12.528 | 0.045 | 0.110 | 0.187 | 0.013 | **0.032** | 0.044 | 0.166 | 0.207 | 0.384 | 0.079 | 0.199 | 0.778 | 0.014 | 0.041 | 0.937 | 5.990 | 12.020 | 28.760 | 0.681 | 1.222 | 2.445 |  | | | 1.228 | 2.758 | 4.216 | 0.245 | **0.500** | 0.868 |
|  |  | **Yes** | 3.243 | 8.075 | 19.035 | 0.101 | 0.191 | 0.312 | 0.040 | **0.079** | 0.141 | 0.140 | 0.360 | 0.721 | 0.098 | 0.216 | 0.570 | 0.057 | 0.113 | 0.280 | 3.882 | 11.275 | 19.025 | 0.907 | 1.403 | 2.862 |  | | | 1.979 | 2.794 | 4.687 | 0.726 | **1.021** | 1.278 |
|  |  | **p value** |  |  |  |  |  |  |  | **0.019** |  |  |  |  |  |  |  |  |  |  |  |  |  |  |  |  |  | | |  |  |  |  | **0.022** |  |
| **PD-L1 expression** |  | **<1%** | 1.748 | 4.857 | 23.075 | 0.052 | 0.129 | 0.274 | 0.043 | 0.080 | 0.110 | 0.121 | 0.370 | 1.094 | 0.051 | 0.201 | 0.625 | 0.032 | 0.109 | 0.247 | 6.235 | 13.410 | 21.043 | 0.525 | 1.323 | 3.317 |  | | | 1.635 | 2.387 | 4.308 | 0.600 | 0.927 | 1.478 |
|  |  | **1%~50%** | 3.501 | 8.662 | 15.490 | 0.095 | 0.126 | 0.141 | 0.038 | 0.043 | 0.089 | 0.208 | 0.547 | 1.610 | 0.096 | 0.146 | 0.441 | 0.011 | 0.091 | 0.105 | 6.764 | 9.687 | 18.010 | 0.896 | 1.037 | 1.620 |  | | | 1.982 | 3.131 | 4.999 | 0.662 | 0.964 | 1.422 |
|  |  | **≥50%** | 2.309 | 4.989 | 8.837 | 0.060 | 0.111 | 0.312 | 0.031 | 0.080 | 0.159 | 0.295 | 0.486 | 0.760 | 0.104 | 0.250 | 0.559 | 0.011 | 0.066 | 0.251 | 3.476 | 13.605 | 19.913 | 0.876 | 1.190 | 2.734 |  | | | 1.724 | 2.537 | 3.879 | 0.381 | 0.643 | 1.158 |
|  |  | **p value** |  |  |  |  |  |  |  |  |  |  |  |  |  |  |  |  |  |  |  |  |  |  |  |  |  | | |  |  |  |  |  |  |
| **Treatment-line** | | **1** | 2.970 | 5.638 | 13.690 | 0.100 | **0.141** | 0.266 | 0.037 | 0.074 | 0.145 | 0.235 | 0.490 | 0.829 | 0.098 | 0.187 | 0.553 | 0.041 | 0.105 | 0.201 | 4.914 | 10.040 | 15.280 | 0.896 | 1.222 | 2.709 |  | | | 1.918 | 2.662 | 4.350 | 0.500 | 0.722 | 1.212 |
|  |  | **≥2** | 1.492 | 4.875 | 17.810 | 0.026 | **0.082** | 0.234 | 0.022 | 0.067 | 0.127 | 0.106 | 0.159 | 0.444 | 0.092 | 0.256 | 0.662 | 0.024 | 0.089 | 0.131 | 7.469 | 9.819 | 20.755 | 0.694 | 1.363 | 2.462 |  | | | 2.026 | 2.429 | 3.649 | 0.713 | 1.089 | 1.451 |
|  |  | **p value** |  |  |  |  | **0.020** |  |  |  |  |  |  |  |  |  |  |  |  |  |  |  |  |  |  |  |  | | |  |  |  |  |  |  |
| **Treatment response** |  | **PD** | 2.925 | 9.905 | 34.255 | 0.045 | 0.109 | 0.128 | 0.037 | 0.065 | 0.248 | 0.112 | **0.210** | 0.518 | 0.198 | 0.397 | 0.706 | 0.033 | 0.144 | 0.316 | 7.011 | 11.310 | 14.425 | 0.454 | 1.547 | 3.248 | 2.126 | 3.014 | 5.001 | 0.787 | 1.070 | 1.278 |  |  |  |
|  |  | **PR** | 2.583 | 7.629 | 11.233 | 0.106 | 0.176 | 0.291 | 0.039 | 0.093 | 0.247 | 0.370 | **0.651** | 1.209 | 0.065 | 0.125 | 0.517 | 0.021 | 0.079 | 0.117 | 3.796 | 8.044 | 20.380 | 0.934 | 1.401 | 2.651 | 1.696 | 2.250 | 4.372 | 0.541 | 0.792 | 1.254 |  |  |  |
|  |  | **SD** | 2.408 | 4.691 | 11.050 | 0.046 | 0.138 | 0.262 | 0.032 | 0.067 | 0.105 | 0.159 | **0.358** | 0.715 | 0.093 | 0.191 | 0.586 | 0.035 | 0.093 | 0.158 | 6.670 | 10.040 | 18.310 | 0.896 | 1.222 | 2.256 | 1.974 | 2.429 | 3.504 | 0.355 | 0.722 | 1.285 |  |  |  |
|  |  | **pvalue** |  |  |  |  |  |  |  |  |  |  | **0.025** |  |  |  |  |  |  |  |  |  |  |  |  |  |  |  |  |  |  |  |  |  |  |

Table S3. Time-dependent ROC analysis of the combined baseline serum levels in association with PFS

| Number of cytokines | Combination | AUC at 1 year | AUC at 2 years | AUC at 3 years |
| --- | --- | --- | --- | --- |
| 2 | IL-4 + IL-5 | 0.583 | 0.507 | 0.734 |
|  | IL-4 + IL-8 | 0.614 | 0.703 | 0.608 |
|  | IL-4 + IL-10 | 0.726 | 0.589 | 0.818 |
|  | IL-4 + TNF-α | 0.640 | 0.638 | 0.910 |
|  | IL-5 + IL-8 | 0.570 | 0.645 | 0.819 |
|  | IL-5 + IL-10 | 0.715 | 0.698 | 0.884 |
|  | **IL-5 + TNF-α** | **0.657** | **0.740** | **0.936** |
|  | IL-8 + IL-10 | 0.707 | 0.626 | 0.824 |
|  | IL-8 + TNF-α | 0.628 | 0.710 | 0.920 |
|  | IL-10 + TNF-α | 0.705 | 0.726 | 0.929 |
| 3 | IL-4 + IL-5 + IL-8 | 0.536 | 0.486 | 0.732 |
|  | IL-4 + IL-5 + IL-10 | 0.718 | 0.621 | 0.854 |
|  | IL-4 + IL-5 + TNF-α | 0.635 | 0.627 | 0.900 |
|  | IL-4 + IL-8 + IL-10 | 0.702 | 0.583 | 0.807 |
|  | IL-4 + IL-8 + TNF-α | 0.613 | 0.619 | 0.911 |
|  | IL-4 + IL-10 + TNF-α | 0.702 | 0.652 | 0.921 |
|  | IL-5 + IL-8 + IL-10 | 0.694 | 0.681 | 0.887 |
|  | IL-5 + IL-8 + TNF-α | 0.625 | 0.716 | 0.922 |
|  | IL-5 + IL-10 + TNF-α | 0.710 | 0.732 | 0.934 |
|  | IL-8 + IL-10 + TNF-α | 0.686 | 0.703 | 0.915 |
| 4 | IL-4 + IL-5 + IL-8 + IL-10 | 0.699 | 0.610 | 0.858 |
|  | IL-4 + IL-5 + IL-8 + TNF-α | 0.608 | 0.624 | 0.930 |
|  | IL-4 + IL-5 + IL-10 + TNF-α | 0.710 | 0.659 | 0.914 |
|  | IL-4 + IL-8 + IL-10 + TNF-α | 0.684 | 0.629 | 0.915 |
|  | IL-5 + IL-8 + IL-10 + TNF-α | 0.691 | 0.706 | 0.909 |
| 5 | IL-4 + IL-5 + IL-8 + IL-10 + TNF-α | 0.692 | 0.650 | 0.922 |

Table S4. Time-dependent ROC analysis of the combined baseline serum levels in association with OS

| Number of cytokines | Combination | AUC at 1 year | AUC at 2 years | AUC at 3 years |
| --- | --- | --- | --- | --- |
| 2 | IL-6 + IL-8 | 0.671 | 0.687 | 0.685 |
|  | IL-6 + TNF-α | 0.547 | 0.565 | 0.537 |
|  | IL-8 + TNF-α | 0.544 | 0.501 | 0.529 |
| 3 | IL-6 + IL-8 + TNF-α | 0.599 | 0.578 | 0.578 |

Table S5. Changes of serum cytokine levels after 2 treatment cycles in patients with different treatment response

| Treatment response | Treatment cycle | Cytokine levels (median, pg/ml） | | | | | | | | | |
| --- | --- | --- | --- | --- | --- | --- | --- | --- | --- | --- | --- |
|  |  | IL-6 | IL-12 | IL-1b | IL-4 | IL-5 | IFN-γ | IL-8 | IL-22 | TNF-α | IL-10 |
| PR + SD | 0 | 7.78 | 0.13 | 0.08 | 0.41 | 0.13 | 0.09 | 9.93 | 1.28 | 2.07 | 0.67 |
|  | 2 | 6.68 | 0.14 | 0.04 | 0.32 | 0.21 | 0.11 | 9.99 | 1.61 | 2.70 | 1.11 |
|  | pvalue |  |  |  |  | **0.0076** | **0.0335** |  |  |  | **0.0003** |
| PD | 0 | 9.91 | 0.1 | 0.06 | 0.19 | 0.28 | 0.13 | 9.51 | 2.18 | 2.66 | 1.12 |
|  | 2 | 17.7 | 0.14 | 0.09 | 0.25 | 1.17 | 0.33 | 6.21 | 2.19 | 4.62 | 3.32 |
|  | pvalue |  |  |  |  |  |  |  |  | **0.0185** |  |
