# Supplementary figures and images for: Prognostic role of serum cytokines level in non-small cell lung cancer patients with anti-PD-1 and chemotherapy combined treatment
Source: Front Immunol. 2024 Oct 22;15:1430301. doi: 10.3389/fimmu.2024.1430301 (PMC11534701; doi:10.3389/fimmu.2024.1430301)

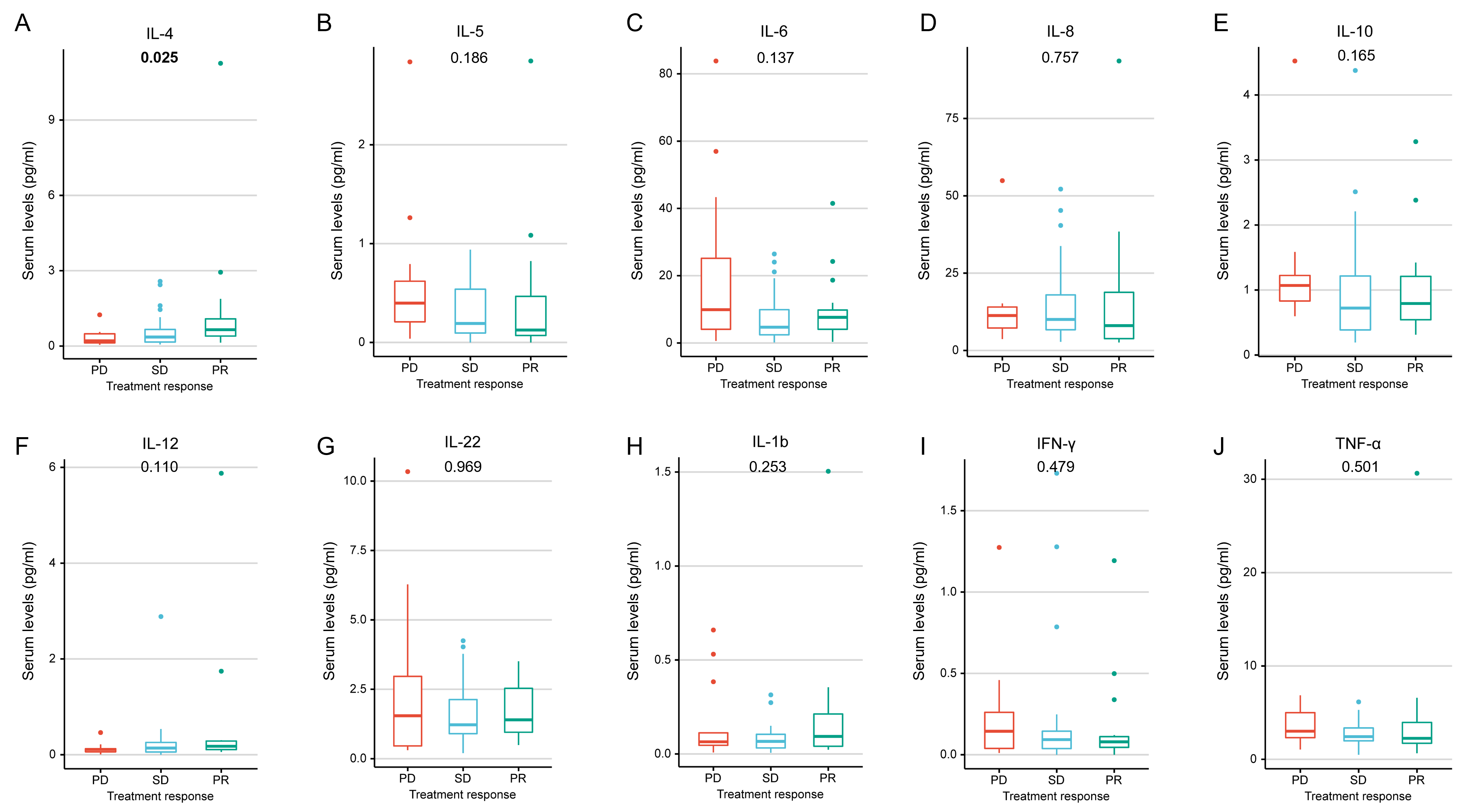

Supplement: Supplementary Figure 1 — Boxplot of serum baseline cytokines among different treatment response categories. [file Image1.tif]

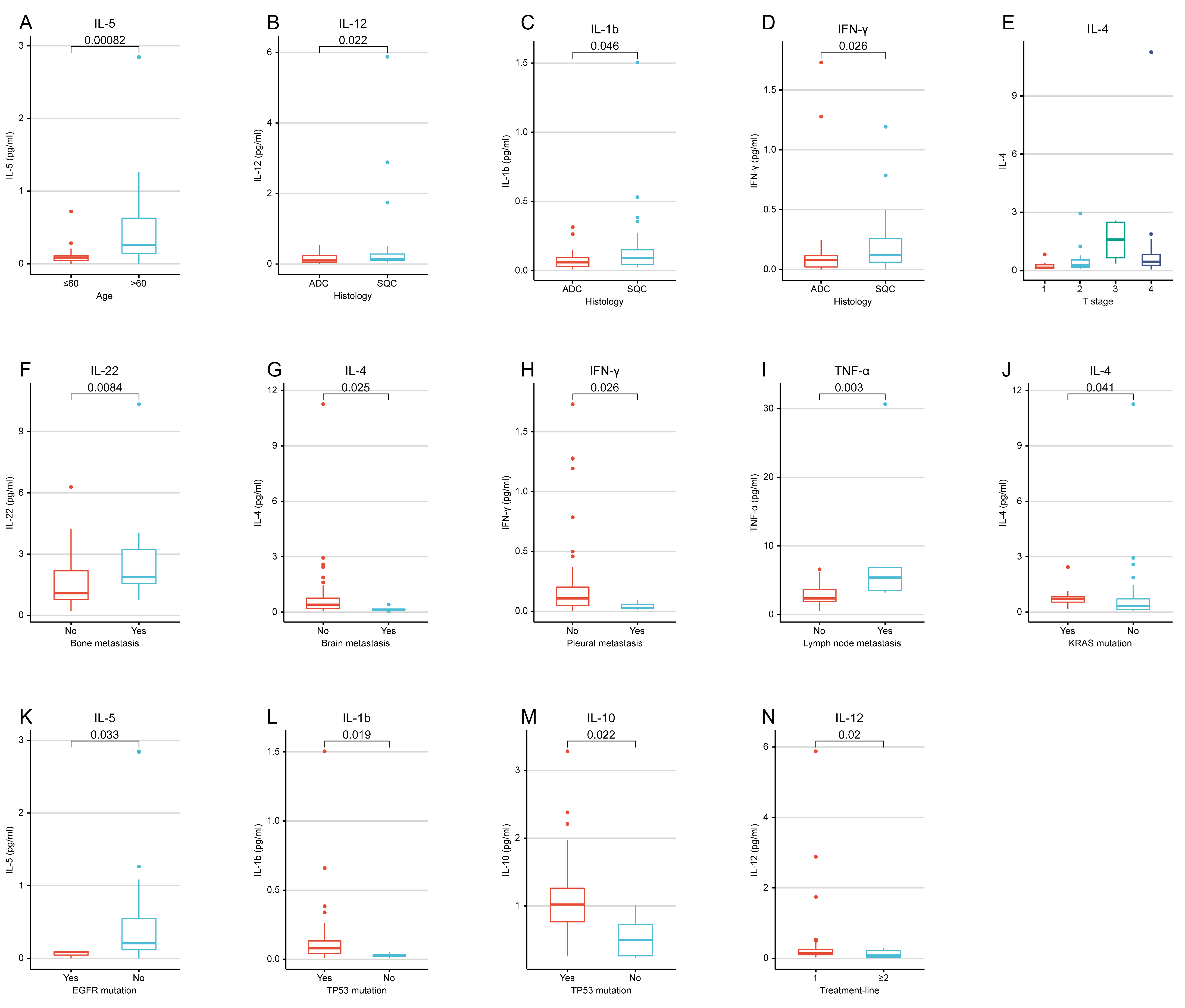

Supplement: Supplementary Figure 2 — Boxplot of serum baseline cytokines among different clinicopathological groups with statistical significance. [file Image2.tif]

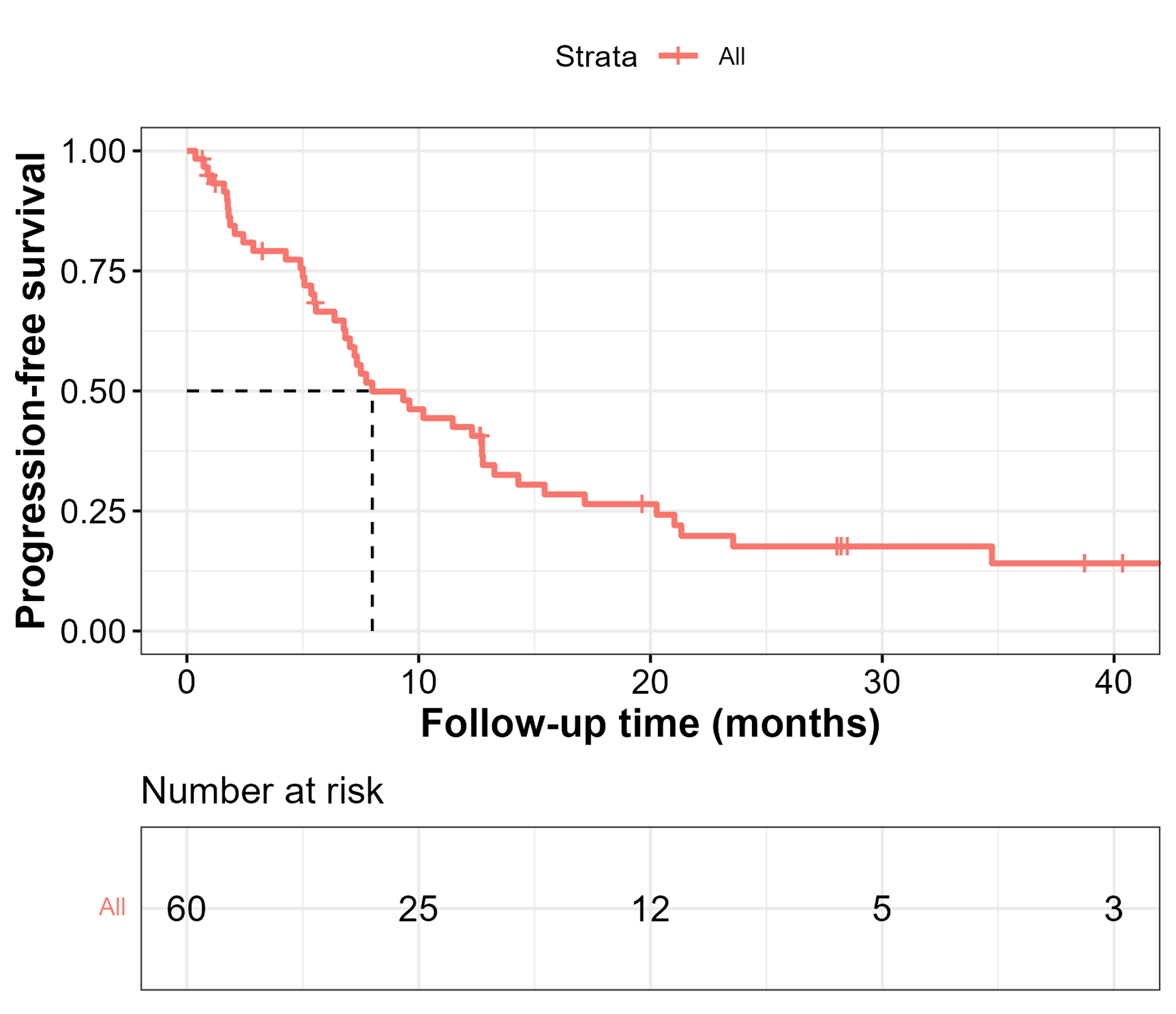

Supplement: Supplementary Figure 3 — Kaplan-Meier analysis of overall PFS of 60 patients receiving chemoimmunotherapy. [file Image3.tif]

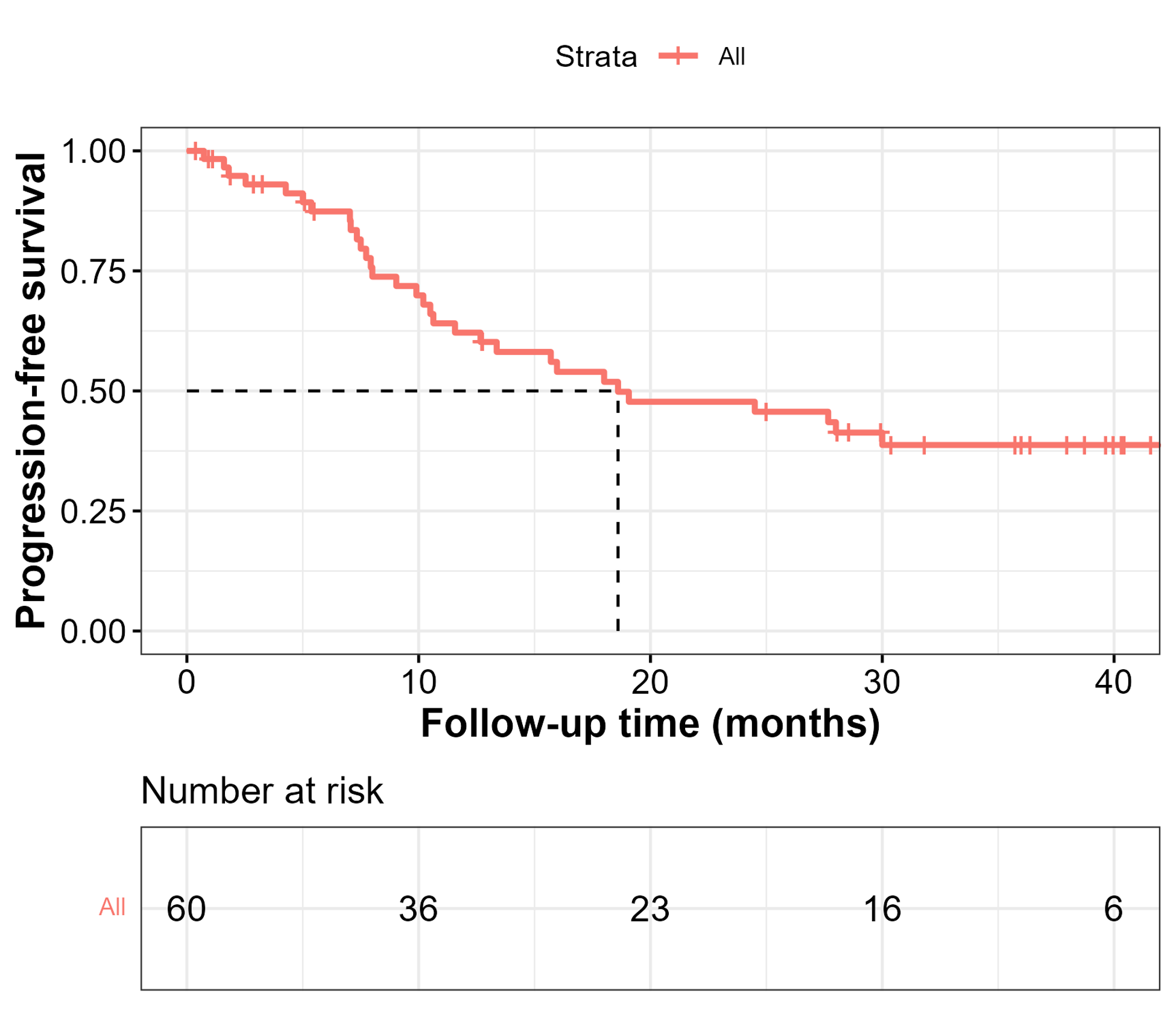

Supplement: Supplementary Figure 4 — Kaplan-Meier analysis of overall OS of 60 patients receiving chemoimmunotherapy. [file Image4.tif]

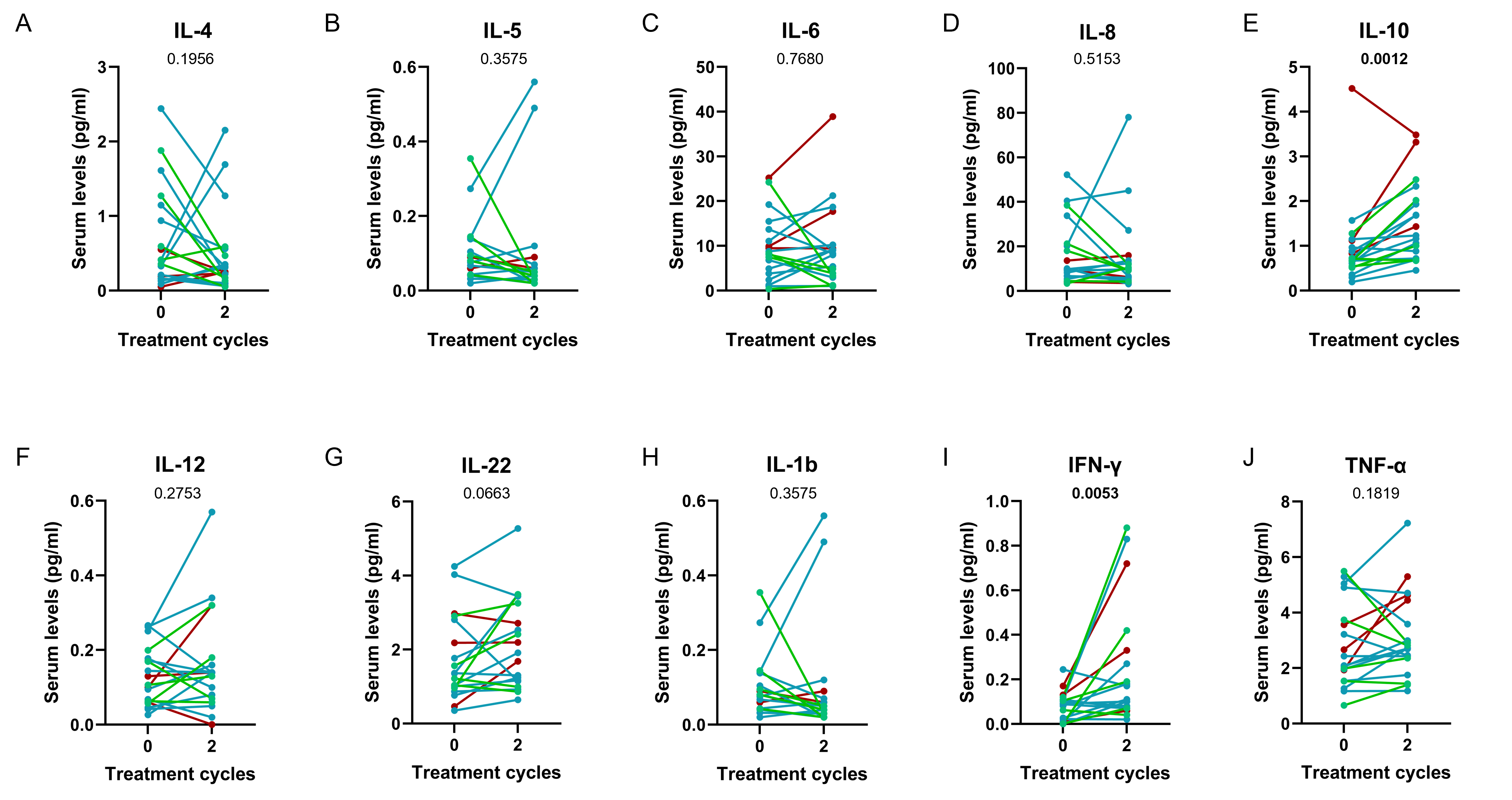

Supplement: Supplementary Figure 5 — Changes of 19 patients’ serum cytokine levels after 2 treatment cycles (red: PD, green: PR, dark cyan: SD). [file Image5.tif]
